# The Agent is Right: When Motor Embodied Cognition is Space-Dependent

**DOI:** 10.1371/journal.pone.0025036

**Published:** 2011-09-23

**Authors:** Claudia Gianelli, Alessandro Farnè, Romeo Salemme, Marc Jeannerod, Alice C. Roy

**Affiliations:** 1 CNRS FRE 3406, Institut des Sciences Cognitives, L2C2, Bron, France; 2 INSERM U1028; CNRS UMR5292; Lyon Neuroscience Research Center, Impact Team, Lyon, France; 3 Université Claude Bernard Lyon, Lyon, France; 4 Dipartimento di Psicologia, Università di Bologna, Bologna, Italy; Yale University, United States of America

## Abstract

The role of embodied mechanisms in processing sentences endowed with a first person perspective is now widely accepted. However, whether embodied sentence processing within a third person perspective would also have motor behavioral significance remains unknown. Here, we developed a novel version of the Action-sentence Compatibility Effect (ACE) in which participants were asked to perform a movement compatible or not with the direction embedded in a sentence having a first person (Experiment 1: You gave a pizza to Louis) or third person perspective (Experiment 2: Lea gave a pizza to Louis). Results indicate that shifting perspective from first to third person was sufficient to prevent motor embodied mechanisms, abolishing the ACE. Critically, ACE was restored in Experiment 3 by adding a virtual “body” that allowed participants to know “where” to put themselves in space when taking the third person perspective, thus demonstrating that motor embodied processes are space-dependent. A fourth, control experiment, by dissociating motor response from the transfer verb's direction, supported the conclusion that perspective-taking may induce significant ACE only when coupled with the adequate sentence-response mapping.

## Introduction

Increasing evidence supports the notion that embodied processes take place while we are either merely observing actions being made by others, or just hearing verbal descriptions of such actions. In this context, neuroimaging findings have highlighted the activity of the motor system during the processing of verbally described actions in the absence of any performed or imagined action [1], [2], [3]. The recruitment of the sensori-motor system during language processing is thought to serve embodied understanding, as it has been shown to impact the subject's motor [4], [5], [6] and perceptual behavior [7], [8]. For instance, when deciding with a backward or forward movement whether a sentence is meaningful or meaningless Glenberg and Kaschak [9] reported faster responses when the direction embedded in the sentence was congruent with the response movement direction (e.g., “You gave the pizza to Andy” & forward movement or “Andy gave you the pizza” & backward movement). In this action-sentence compatibility effect (ACE) participants are called into action directly: they seem to read “You gave…” as “You moved away from your body”, thus acting from a strictly first-person perspective.

In everyday life, we often behave as agents or recipients of actions, and both roles are intimately linked to the experience of having a first-person perspective, a point of view on the world that is only ours. We can take somebody else's perspective, though and we can experience actions from a third person perspective, particularly through language. Pronouns and nouns may shift perspective in a way that is very important for our social interactions, as the more we are able to grasp different aspects of actions and situations, the more we will be socially adapted. In a straight-forward view of embodied cognition, transfer sentences would automatically activate the correspondent transfer actions, their effectors and possibly their kinematics. However, it has been made clear that not only verbs, but other parts of sentences, like pronouns and nouns, can affect motor behavior [10], [11].

Despite the obvious relationships between embodied processing and perspective-taking, the issue of linguistic perspective has rarely been investigated in the field of embodied cognition, whilst some linguistic studies have addressed this question [12]. As recently stressed by Zwaan [13], perspective is a challenge for embodied theories of language comprehension (pp. 20–21), since they need to give account of perspective taking and at the same time are questioned by the behavioral effects of perspective itself. Does any point of view induce the same, automatic motor resonance or does it depend on the situation that language contributes to describe? A recent study on pronoun-induced perspective by Brunyé and coworkers [14] found that an internal (i.e., embodied) perspective is assumed when using the pronoun “You”, but not “He”, affecting the comprehension of simple narratives.

Here we asked whether the embodied processing of action sentences would produce detectable motor effects when participants are required to take someone else's perspective. To this purpose, we manipulated different features of perspective taking and developed a novel version of the well established Action-sentence Compatibility Effect (see also [15]) in which subjects are no longer involved directly into action, but have to put themselves in an avatar's shoes. We predicted that participants' motor performance would be affected according to the possibility of taking or not a first person perspective. In particular, we predicted that only the assumption of a 1^st^ person perspective on action would produce a significant ACE phenomenon, whereas the assumption of an external, 3^rd^ person perspective, would not.

## Experiment 1

In a first experiment, we aimed at validating the ACE in French, as it is endowed with different dative constructions with respect to English. As in the original experiment by Glenberg and Kaschak [9], all the sentences used the pronoun YOU, both as the agent or the recipient of actions, thus directly calling the participants into action. Similarly to the classical ACE paradigms, we required participants to evaluate whether sentences were meaningful or meaningless by moving a joystick away or towards their body.

### Methods

#### Participants

Thirty-two students of Lyon University participated in experiment 1 for which they gave their informed consent. All were right-handed, native French speakers with normal or corrected-to-normal vision and were naive as to the purpose of the experiment. For this, as well as for the following experiments, all participants gave their verbal informed consent to participate in the study, which was approved by the review board of the INSERM U864 (now U1028) ethics committee.

#### Stimuli

We modified the original set of stimuli of Glenberg and Kaschak [9] by following the work by Borregine and Kaschak in which imperative sentences were eliminated [15]. Stimuli consisted of transfer sentences, implying the action of giving/receiving something, either concrete or abstract. Sentences were composed by a noun/personal pronoun to indicate agent/recipient of action, a verb in the past tense and a noun to indicate the object transferred. The final set of stimuli (see Supporting Information, Appendix S1) in French comprised: 40 sentences in the form “You gave x to Louis” (“Tu as donné x à Louis”) divided into 20 abstract (“Tu as donné une chance à Louis”, You gave a chance to Louis)) and 20 concrete sentences (“Tu as donné un livre à Louis”, You gave a book to Louis). The pool of sentences was constituted by 40 additional sentences in the form “Louis gave you x” (“Louis t'a donné x”), similarly divided into 20 abstract and 20 concrete sentences.

The set was completed by 40 non-sense sentences (abstract and concrete) of the first form (“Tu as lance un crocodile à Louis”, You throw a crocodile to Louis), and 40 non-sense sentences of the second form. Stimuli were displayed on a computer screen and were randomly repeated into 2 blocks, for a total of 320 trials.

#### Procedure

The experiment took place in a sound-attenuated booth. Participants sat in front of a computer screen holding with their right hand a joystick located in front of them. The distance between the participant's head and the screen was about 70 cm.

Each trial started by displaying a central fixation cross, then a sentence was presented until the participant started moving the joystick, with a response time limited to 4000 ms. Participants were instructed to read the sentence and to move the joystick (away or towards the body) to respond as to whether the sentence made sense or not, as soon as they could. Participants were randomly assigned to one of two possible conditions, starting with a response away for YES and towards for NO, or the reverse, and the order was reversed in the second block. The response was recorded as soon as the joystick reached a predetermined extent of linear displacement, thus measuring the response time (i.e., the time between sentence onset and beginning of movement, ‘reading time’ in [9]). The sentence disappeared once this response threshold was passed.

#### Data analysis

Data on response times for correct trials were analyzed for each participant. Times beyond ±2,5 standard deviations from average were trimmed for each condition separately for each participant. Final movement direction (away, toward the body) was checked for each trial, to verify the accuracy of participants' movements (i.e. that they did not start moving in a direction and then changed). The mean error rate was of 7% and errors were evenly distributed among the different conditions. As errors analyses revealed no speed-accuracy tradeoff, we focused on RTs.

We applied a repeated measures ANOVA to the mean RTs for each subject in each condition with type of verb (abstract/concrete), role in sentence (agent/recipient), and movement direction (away/toward) as within-subject variables. The effect size was also calculated for each significant variable (η^2^).

### Results

**Figure 1:**
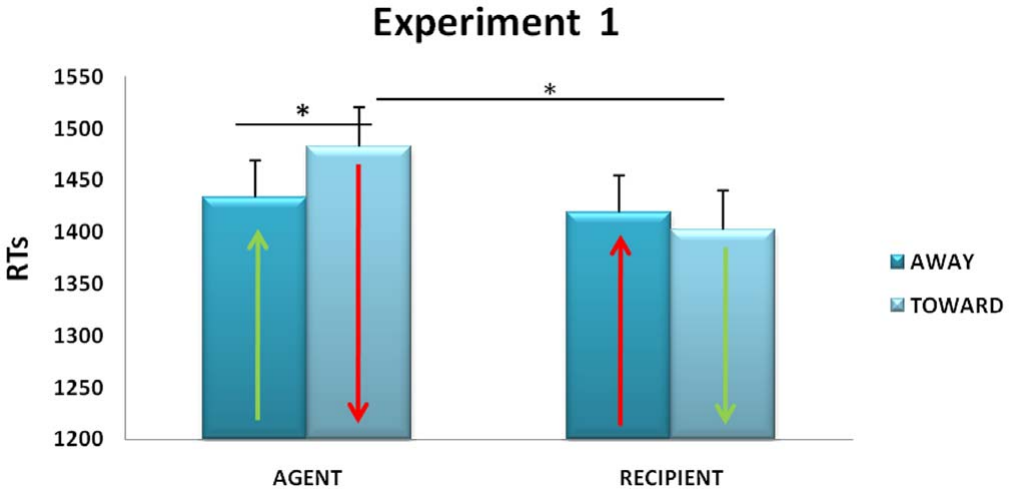
Mean Response Time values for the French version of the ACE paradigm, as revealed by Experiment 1. The arrows indicate response movement direction. The green arrows correspond to the compatible conditions, agent-away and recipient-toward. The red arrows correspond to the incompatible conditions.

The data on response times (RTs) showed a main effect of verb type, as subjects responded faster to concrete than abstract sentences (*F*(1,31) = 41.73, *p*<0.001, η^2^ = 0.7). A main effect of role in sentence was also present (*F*(1,31) = 6.81, *p*<0.05, η^2^ = 0.1): when the participant (YOU in the sentences) was the agent it took her longer to respond as compared to when she was the recipient. Crucially, the interaction role x movement direction was significant (*F*(1,31) = 4.52, *p*<0.05, η^2^ = 0.1), which means the ACE was present. Newman-Keuls post hoc test confirmed that participants in the role of agent answered faster with a compatible movement (i.e., away from their body) than an incompatible movement (i.e., toward their body; *p*<0.05; see Figure 1). In the same line, participants produced faster toward response movements when they were in the role of recipients than agents (*p*<0.01; Figure 1). The interaction involving type of verb was not significant, but for comparative purposes with other studies, separate performances on abstract and concrete sentences are illustrated in Appendix S4.

### Discussion

This first experiment validated our French version of the ACE. Movement directions compatible with the role in the sentence (i.e., away/agent & toward/recipient) resulted in shorter response times, particularly in the agent condition (see Figure 1). The worst performance was observed when participants took on the agent role, but had to respond with an incompatible movement direction toward their body, this condition yielding to a significant increase in response times compared to both the agent-away and the recipient-toward conditions. Overall, these results indicate that participants behaved as if the pronoun “you” in the sentences was referring to themselves, and thus took a first person perspective while performing the task. It is worth noting that the presence of a significant ACE with the use of a joystick constitutes a methodological extension of previous studies, in which button-press responses were used. Joystick responses involve an additional component of object-manipulation (the joystick is always in the hands of the participant) to the traditional away-towards the body movements.

Besides extending the general principle of the ACE phenomena to a language with dative structures differing from English, the findings of Experiment 1 suggest that the ACE may be selectively obtained for the agent role. Nevertheless, when assuming the recipient role, participants were more likely to show facilitation for movements directed toward them. This experiment leaves open the question as to whether similar embodied processing would occur when participants are asked to take a third person's perspective. Experiment 2 and 3 were designed to directly assess this question.

## Experiment 2

In the second experiment, our purpose was to investigate whether the ACE is strictly dependent or not upon taking a first-person perspective. To this aim, we modified the sentences of Experiment 1 by introducing the names of LOUIS and LEA as actors of a dyadic transfer interaction, in the form “Louis gave x to Léa”. Participants were no longer called into action directly, but they were asked to assume the perspective of one of the actors (Louis for males, Léa for females) and perform the task as if they were him/her.

### Methods

#### Participants

Thirty-four students of Lyon University participated in experiment 2. The same criteria as in Experiment 1 were followed.

#### Stimuli

The pronoun YOU was substituted by using two external actors, Louis and Léa in the same set of sentences used in experiment 1. The structure and number of stimuli were otherwise identical (see Appendix S2 in the Supporting Information).

#### Procedure

The procedure was the same as in Experiment 1. Here, participants were additionally asked to take one of the actors' perspective (third person) and to read the sentences as if they were one of the two actors, assigned depending on participants' gender (see Appendix S5 for a complete translation of the instructions). At the end of the experimental session each participant filled a questionnaire in order to self-evaluate her performance during the perspective-taking and sensibility judgment tasks. A list of 16 statements was presented and each participant was required to indicate her agreement on a 14 cm horizontal line, where the extreme left indicated “I do not agree at all” and the extreme right indicated “I completely agree”. The full questionnaire is available in the Supporting Information (see Appendix S3).

#### Data analysis

The mean error rate was 4%, errors without any speed-accuracy tradeoff. Data on response times for correct trials were thus analyzed for each participant as in experiment 1.

In addition, the mean values of agreement for each item of the questionnaire were computed and then analyzed.

### Results

**Figure 2:**
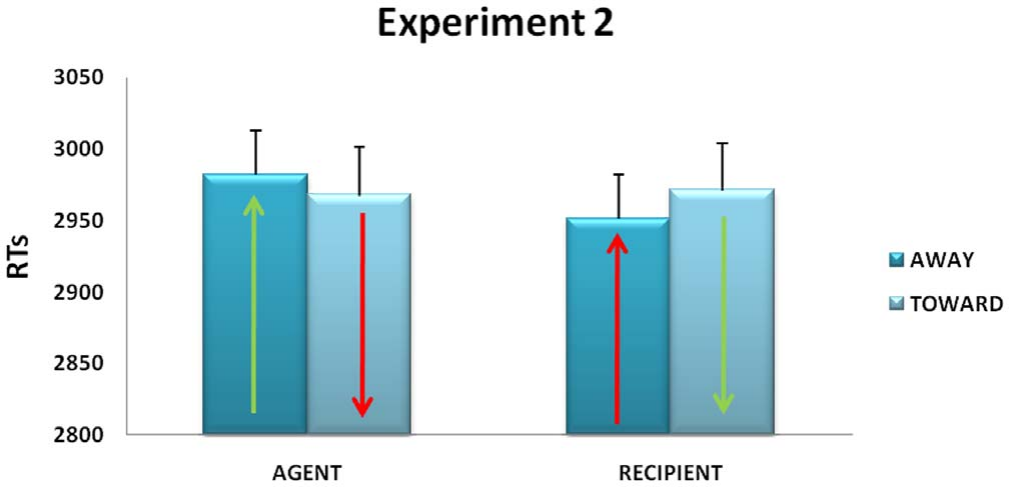
Mean Response Time values for the third person perspective version of the ACE paradigm, as revealed by Experiment 2. Conventions as in Figure 1. Note the longer response time and the trend to an opposite pattern with respect to Experiment 1.

As in Experiment 1, concrete verbs yielded to faster responses than abstract verbs (*F*(1,33) = 8.76, *p*<0.01, η^2^ = 0.6). However, the interaction role x movement (i.e., the ACE), was not significant (*F*(1,33) = 3.043, *p* = 0.09). There was actually a non significant trend to an opposite pattern with respect to the classical ACE (see Appendix S4 for separate illustration of abstract and concrete sentences results): responses for the recipient-away condition and agent-toward condition tended to be faster (see Figure 2). As indicated by the average response time, overall longer with respect to the first experiment, and by the questionnaire results participants correctly performed the perspective-taking task and showed a good comprehension of the task itself.

To statistically compare experiments 1 and 2, we performed an ANOVA with experiment (1, 2) as between-subject variable and type of verb (abstract/concrete), role in sentence (agent/recipient), and movement direction (away/toward) as within-subject variables. The analysis showed a significant main effect of experiment. In support to the idea that the perspective taking task was correctly performed, RTs were longer in experiment 2 than in experiment 1 (*F*(1,63) = 229.3, *p*<0.01). As expected, the main effect of Verb Type was also significant (*F*(1,63) = 40, *p*<0.01, η^2^ = 0.4), with concrete sentences yielding to faster RTs than abstract ones. Crucially, the interaction Experiment X Role X Movement was also significant (*F*(1,63) = 12.5, *p*<0.05, η^2^ = 0.2). Post-hoc test confirmed the significant ACE in experiment 1, specifically for the Agent role (*p*<0.01), and its absence in experiment 2, for all conditions alike (all *p_s_*>0.05).

### Discussion

When sentences did not imply the participants to take a first person perspective, as the transfer action occurred between two external actors (Louis and Léa), the ACE was no longer observed, despite using the very same set of sentences. In other words, shifting the participant's perspective, from a first to a third person, was sufficient to prevent the action-sentence compatibility effect to occur. As a corollary, in order to induce a, behaviorally effective motor perspective it is not sufficient to ask participants to act “as if” they were another person. The absence of direct motor effects does not exclude that sentences may cause a perceptual embodiment. However, it is worth noting that in experiment 2 there is no automatic activation of transfer direction, as typically involving a relationship between an agent and a recipient with specific movements away or towards the body. We hypothesize that the ACE did not occur because participants were unable to put themselves “in action”, to assume an effective motor perspective about the sentences they were reading. We thus performed a third experiment in which a spatial anchor was given to position the avatars' location and possibly render the motor perspective taking more effective.

## Experiment 3

The results of Experiment 2 suggest that the motor effects of processing linguistic actions are constrained by the participant's perspective. Trying to act as if we were in another person's shoes does not recruit the same network we recruit when acting in a first person perspective. However, one might argue that “embodied” processes might be prevented when one is supposed to assume the perspective of a *disembodied* character, as it was the case for our second experiment where the two characters were abstract and lacked even the minimal feature of spatial location (see [16], [17], [18]). We reasoned that adding a virtual “body”, in the form of a simple spatial anchor, will allow the participant to know “where” to put herself in space when taking Léa's perspective. Following this rationale, in a third experiment we tested the hypothesis that adding spatial information to the perspective-taking manipulation would create the conditions for the ACE to be re-instated.

### Methods

#### Participants

Thirty-four students of Lyon University participated in experiment 3. The criteria were the same as in experiment 1 and 2.

#### Stimuli

**Figure 3:**
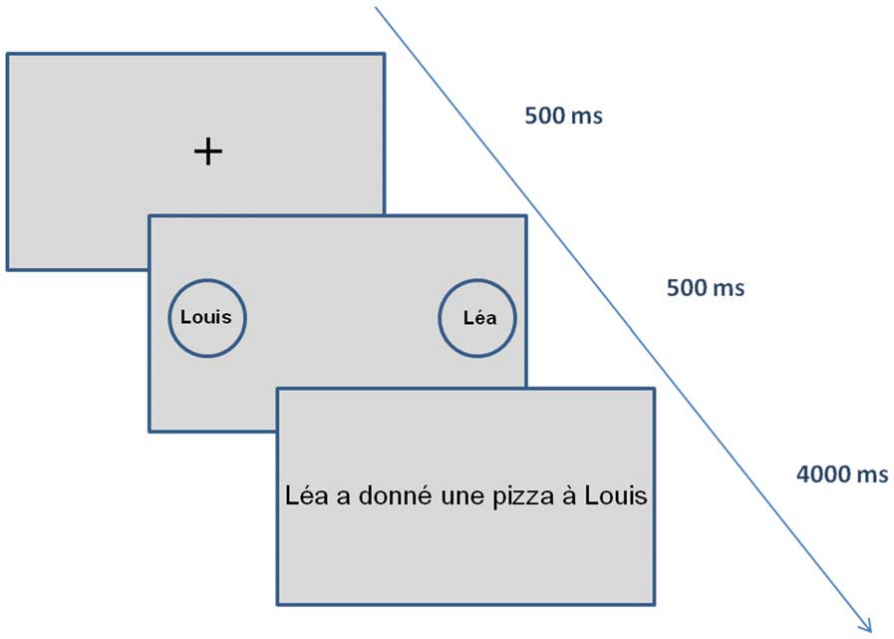
Temporal sequence of the display used in Experiment 3, adding a place-holder for each actor.

The same set of stimuli of experiment 2 was used. In order to enable a spatial anchoring in the perspective taking, each trial started with the presentation of the spatial position of Louis and Léa for 500 ms before sentence onset (see Figure 3). The names “Louis” and “Léa” were presented within two circles located on the right or the left of the screen and the actors' position was totally task-irrelevant. Left and right positions were used in order to maintain the two names orthogonal to the response direction, as the sentences already were. For this reason, we avoided the use of other positions (namely, on the upper and lower part of the screen), which would have induced an additional dimension with respect to the participant's body and moving hand, and thus a potential confound. Furthermore, we decided to reproduce a spatial anchor that could map the linguistic structure of French sentences, where the agent is typically on the left and the recipient on the right. In this sense the spatial anchor could be directly mapped onto the position of the two actors in the sentence, thus producing congruent and incongruent positions that could modulate the ACE if a perspective is effectively taken.

Stimuli were divided into four blocks of trials, which randomly assigned one character (Louis or Léa) to a spatial position (left or right) and one movement direction (away or toward) to a response (sensible or not). The final design comprised 2 spatial positions (left/right), 2 roles in sentence (agent/recipient), 2 verbs (abstract/concrete), and 2 movement directions (away/toward).

#### Procedure

The procedure and questionnaire were the same as in Experiment 2. In addition, participants were instructed to look at the spatial positions of Louis/Léa, then to read the sentence and perform the task as if they were one of the two actors (exactly as in experiment 2).

#### Data analysis

The mean error rate was 5%: Again, due to the absence of any speed-accuracy tradeoff, we focused on RTs. Data on response times for correct trials were analyzed for each participant as in experiment 1 and 2. We applied a repeated measures ANOVA to the mean response times of the participants, with spatial position (left/right), type of verb (abstract/concrete), role in sentence (agent/recipient), and movement direction (away/toward) as within-subject variables. The effect size was also calculated for each significant variable (η^2^). The mean values of agreement for each item of the questionnaire were also computed and analyzed as in Experiment 2. In addition, a paired t-test was applied to compare the level of agreement across experiments 2 and 3.

### Results

**Figure 4:**
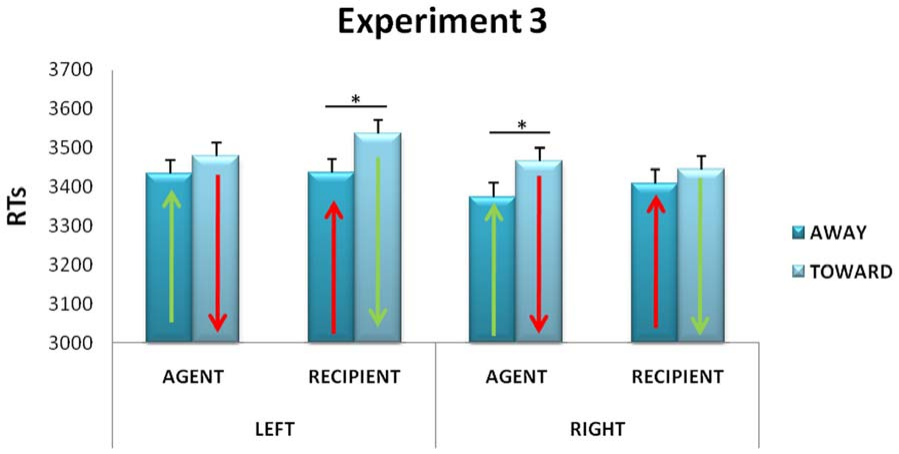
Mean Response Time values for the spatialized version of the third person perspective of the ACE paradigm, as revealed by Experiment 3, as a function of the left and right spatial position of the participant's avatar location. Conventions as in Figure 1.

As in the first two experiments the analyses revealed shorter response times for sentences endowed with a concrete verb with respect to an abstract verb (*F*(1,33) = 32.53, *p*<0.001, η^2^ = 0.5, see Appendix S4 for graphics representing abstract and concrete results separately). Spatial position (right/left) presented a marginally significant effect (*F*(1,33) = 3.63, p = 0.06, η^2^ = 0.1), right spatial position tending to be associated with shorter response times. Crucially, the interaction between spatial position, role and movement direction was significant (*F*(1, 33) = 4.97, *p*<0.05, η^2^ = 0.05). Newman-Keuls post-hoc test revealed a compatibility effect in the case of right spatial position (see Figure 4). When participants assumed the agent role, away responses were faster than toward ones (*p*<0.01). This confirms the stronger compatibility effect for agent compared to recipient role we found in the first experiment. For the left spatial position (Figure 4), while the compatibility effect for the agent role tended to be maintained without reaching significance, the recipient condition showed a significant effect: away movements were faster than toward ones (*p*<0.01).

**Figure 5:**
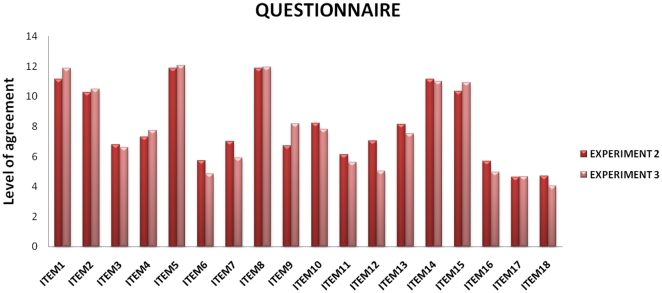
Mean values of agreement in experiments 2 (third person perspective without spatial localization) and 3 (third person perspective with spatial localization) for each item of the questionnaire. Even if the presence of place holder directly affected the response to the behavioral task, the ratings of perspective taking agreement were not different across experiments 2 and 3. See in particular item 3 “ It seemed difficult to me to perform movements as if I were Léa/Louis” (t = −0.12, p = ns), item 4 “It was really easy for me to perform movements as if I were Léa/Louis” (t = 0.4, p = ns). item 7 “I felt immediately in Léa/Louis' shoes” (t = −1.2, p = ns), item 9 “I had difficulties in taking Léa/Louis' perspective” (t = 1.32, p = ns), item 11 “The point of view of Léa/Louis immediately became mine” (t = −0.5, p = ns), item 2 “I have correctly understood my perspective” (t = 0.25, p = ns).

Remarkably, participants' response to the questionnaire suggested they were not (consciously) influenced by the actor's place-holder. In particular, they did not show any explicit preference, or strategy, for the left or right spatial position to perform the perspective task (mean agreement for left and right 4.83 cm and 4.66 cm, respectively). Noteworthy, the agreement in the two experiments did not differ for the crucial items regarding the perspective taking task (see detailed results in Figure 5).

### Discussion

In Experiment 3, while the sentence and the perspective taking tasks were identical to Experiment 2, the ACE was observed again, with a specific pattern dependent on the spatial position of the participant's avatar. On the one hand, when participants took on the role of agent, the compatible response direction (i.e., away) was generally executed faster than the incompatible one, this effect being clear when the place-holder for the character's position was located on the right. On the other hand, when participants took on the recipient role, the incompatible response direction (i.e., away) was generally executed faster than the compatible one (inverse ACE, see [15] for discussion about a possible reversal of the effect depending on response timing), this effect reaching significance only when the character's place-holder was on the left. It is worth noticing that we did not observe the compatibility between agent and left position that we could expect on the basis of words order, the subject/agent being the first word of the sentence. These results suggest that participants' behavior was influenced by two different interacting biases. A first bias would be the tendency to assume and embody the agent more than the recipient role. Somewhat by definition, asking participants to perform movements is somehow already asking them to behave as agents. The second bias would consist in placing themselves on the right of a scene. According to these two biases, the strongest action-sentence compatibility effects occurred when participants took on the role of agent on the right and the ACE was re-instantiated even from a third person perspective. According to the same biases, the strongest incompatibility effects (and inverse ACE) were observed when participants had to assume the role of recipient on the left. The bias to place ourselves as agents on the right of a scene is reminiscent of the right-left bias of western cultures [19], [20]) according to which we tend to attribute agency to actors located on the left when we observe a scene (i.e., to those who place themselves on the right of the scene).

## Experiment 4

The results of experiment 3 suggest that a spatial anchor is capable to restore the ACE under specific constraints and with a pattern that is different for the left and right spatial positions. While at first sight it might seem puzzling that participants could embody a verbal transfer by performing movements perpendicular to the avatars, this is precisely the pattern one would expect if the place-holder were effective in enabling participants to embody the assigned third-person perspective, and were thus reading the sentence from a shifted, first-person perspective. To control for this interpretation, we conducted a fourth experiment with exactly the same stimuli and procedure of experiment 3, but using left-right, instead of back and forth response movements. We reasoned that, in such conditions, no consistent ACE should be observed because the left-right response movement (from the effectively taken actor's perspective) is now incompatible with the direction of giving-receiving implied by transfer sentences.

### Methods

#### Participants

A total of forty-two subjects participated in experiment 4. All were students of Lyon University enrolled according to the same criteria as in experiments 1, 2 and 3. They were constituted by two subgroups of eighteen and twenty-four participants, who performed slightly different versions of the experiment (see below).

#### Stimuli

A subgroup of 18 subjects performed the experiment by using exactly the same set of stimuli and design of experiment 3. Since, again, no interaction with verb type was found on a preliminary analysis, an additional subgroup of 24 subjects performed the experiment by using only the sub-set of stimuli corresponding to the concrete verbs of experiment 3, which were thus analyzed on a total of forty-two participants.

#### Procedure

The procedure was the same as in Experiment 3, expect that participants were instructed to move the joystick to the left or to the right.

#### Data analysis

The mean error rate was of 5%, and no evidence of speed-accuracy tradeoff was present. Data on response times for correct trials were thus analyzed for each participant as in experiment 3. We first applied on the smaller group (N = 18) a repeated measures ANOVA to the mean response times of the participants, with spatial position (left/right), type of verb (abstract/concrete), role in sentence (agent/recipient), and movement direction (left/right) as within-subject variables. The effect size was also calculated for each significant variable (η^2^). The mean values of agreement for each item of the questionnaire were also computed and analyzed as in Experiment 3. Secondly, the same ANOVA, except for verb type (only concrete) was run on the overall sample (N = 42). In addition, a paired t-test was applied to compare the level of agreement across experiments 3 and 4.

### Results

No significant main effect, either of Verb Type or Spatial Position, was present.

The crucial interaction Spatial Position X Role X Movement was far from significance (*F*(1,17) = 0.06, *p* = 0.81), thus showing no specific ACE pattern depending on spatial position or movement type. Re-analyzing the data from experiment 3 with the same number of subjects (i.e., a smaller sample in which N = 18) already showed a trend to significance (*p* = 0.1). To avoid any risk of a Type II statistical error, we performed an ANOVA with spatial position (left/right), role in sentence (agent/recipient), and movement direction (away/toward) as within-subject variables by adding the results of the larger subgroup (total N = 42). This ANOVA revealed that the interaction testing the ACE, namely Spatial Position X Role X Movement, was still very far from significance (F(1,41) = 0.34, p = 0.57). Similar to experiment 3, the questionnaire data did not show any explicit difference for the left and right spatial position of the avatar (t = 1.7, p = ns). Interestingly, the level of agreement for the crucial items regarding perspective did not significantly differ in experiment 4 as compared to experiment 3 (item 6 “It was easier to take Léa/Louis's perspective when he/she was on the left”, t = −0.2, p = ns; item 17 “When Léa/Louis was right, it was easier to take her/his perspective”, t = 1.7, p = ns) suggesting that participants did not show any explicit preference for the left or right spatial position of the avatar, regardless the compatibility of this position with the required motor task.

### Discussion

In the fourth experiment, the use of response movements parallel to the avatars' spatial position, but orthogonal to the direction of transfer action when taking the avatar's perspective, failed to produce any significant ACE. The only, but critical difference between experiment 3 and 4 was the direction of the response movement. In experiment 3, the back and forth movement was compatible with a transfer action experienced by the participants, while in experiment 4 the left-right movement direction was no longer compatible with a transfer action. The partially restored ACE in the third, but not the fourth experiment, clearly suggests that avatars' spatial position may induce significant motor effects only when coupled with the adequate sentence-response mapping. The absence of effect in experiment 4 reinforces the idea that the ACE depends on bodily/spatial constraints: the spatial mapping facilitates the third-person perspective taking and has therefore to be accompanied by a response direction compatible with the newly embodied perspective. Even if congruent with the avatar's positions, the use of left-right movements does not produce a significant motor effect, precisely because it does not map the typical transfer action as experienced when embodying someone else's perspective.

### General discussion

A wealth of data supports the embodied cognition theory, according to which, the processing of verbs and sentences describing actions recruits the motor system and, as such, may impact our motor behavior. Up to now, the studies dealing with sentences have always used pronouns directly calling the participants into action, such as in “You kicked the ball”. The question we addressed through this series of experiments was if one would still recruit her motor system (as seen from overt behavior) to process a sentence in which she is no longer present as a first person, but asked to take someone else's perspective (Beth kicked the ball). To this aim, we manipulated the paradigm known to induce action-sentence compatibility effect (ACE), in which participants are required to judge whether sentences describing a (concrete or abstract) transfer from an agent to a recipient made sense or not. Results from a series of four experiments provide substantial novel insights on how the ACE can be manifest and modulated by perspective taking. Overall, our findings suggest that the Action-sentence Compatibility Effect is not automatic and mandatory, being far more flexible of what it was supposed to be. In the first experiment we extended the ACE to French transfer sentences. In the second experiment, we manipulated perspective-taking by introducing two external actors and asking participants to perform the task “as if they were” one of them. In this case, the ACE was not observed, demonstrating that an effective first-person perspective is necessary to induce ACE phenomena, whereas processing a sentence with a third person perspective does not impact subject's motor behavior. We argue that in this experiment perspective-taking did not occur at a motor level because participants were unable to shift their bodily position in the space of action. Indeed, when we allowed participants to position themselves in the space from which the action takes place (Experiment 3), the ACE phenomena were observed again, though partially. This finding clearly indicates that having a place in space, a “body”, is a necessary step to enable such motor embodiment processes. This conclusion is supported by previous studies [21] showing that compatibility approach-avoidance effects depend on how people represent themselves in space. Finally, our fourth experiment, highlighted the fact that motor embodiment depends upon the possibility of acting “as if” in a first-person perspective: having a spatial position for our bodily self is necessary, but not sufficient, as the direction of our response movement needs to be performed in reference to the self to reflect an sentence embodiment.

To conclude, we suggest that the motor effects of language processing are constrained by the perspective of a specific agent with a specific body position in space. When the body of the participant is the only reference available for movements, shifting perspective is sufficient to preclude any detectable motor effect using the ACE phenomenon. Adding a spatial anchor to perspective taking makes the motor effects reappear (under specific constraints), suggesting that spatially localizing ourselves allows embodying somebody else's perspective.

## Supporting Information

Appendix S1 **Set of stimuli used in** **experiment 1** **.**
(DOC)

Appendix S2 **Set of stimuli used in** **experiment 2** **,** **3** **and** **4** **.**
(DOC)

Appendix S3 **Example of questionnaire used for debriefing after** **experiments 2** **,** **3** **and** **4** **.**
(DOC)

Appendix S4 **Separate performances for abstract and concrete sentences in all experiments.**
(DOC)

Appendix S5 **Translation of complete instructions for** **experiment 2** **.**
(DOC)
