# Supplementary material for: The Agent is Right: When Motor Embodied Cognition is Space-Dependent
Source: PLoS One. 2011 Sep 23;6(9):e25036. doi: 10.1371/journal.pone.0025036 (PMC3179480; doi:10.1371/journal.pone.0025036)
Supplement: Appendix S1 — Set of stimuli used in experiment 1 . (DOC) [file pone.0025036.s001.doc]

| | **1** | | --- | | **2** | | **3** | | **4** | | **5** | | **6** | | **7** | | **8** | | **9** | | **10** | | **11** | | **12** | | **13** | | **14** | | **15** | | **16** | | **17** | | **18** | | **19** | | **20** | | **21** | | **22** | | **23** | | **24** | | **25** | | **26** | | **27** | | **28** | | **29** | | **30** | | **31** | | **32** | | **33** | | **34** | | **35** | | **36** | | **37** | | **38** | | **39** | | **40** | | **41** | | **42** | | **43** | | **44** | | **45** | | **46** | | **47** | | **48** | | **49** | | **50** | | **51** | | **52** | | **53** | | **54** | | **55** | | **56** | | **57** | | **58** | | **59** | | **60** | | **61** | | **62** | | **63** | | **64** | | **65** | | **66** | | **67** | | **68** | | **69** | | **70** | | **71** | | **72** | | **73** | | **74** | | **75** | | **76** | | **77** | | **78** | | **79** | | **80** | | **81** | | **82** | | **83** | | **84** | | **85** | | **86** | | **87** | | **88** | | **89** | | **90** | | **91** | | **92** | | **93** | | **94** | | **95** | | **96** | | **97** | | **98** | | **99** | | **100** | | **101** | | **102** | | **103** | | **104** | | **105** | | **106** | | **107** | | **108** | | **109** | | **110** | | **111** | | **112** | | **113** | | **114** | | **115** | | **116** | | **117** | | **118** | | **119** | | **120** | | **121** | | **122** | | **123** | | **124** | | **125** | | **126** | | **127** | | **128** | | **129** | | **130** | | **131** | | **132** | | **133** | | **134** | | **135** | | **136** | | **137** | | **138** | | **139** | | **140** | | **141** | | **142** | | **143** | | **144** | | **145** | | **146** | | **147** | | **148** | | **149** | | **150** | | **151** | | **152** | | **153** | | **154** | | **155** | | **156** | | **157** | | **158** | | **159** | | **160** | | | **Tu as adressé un courrier à Léa** | | --- | | **Tu as cédé ta place à Léa** | | **Tu as chanté une chanson à Léa** | | **Tu as communiqué le message à Léa** | | **Tu as confié ton secret à Léa** | | **Tu as consacré du temps à Léa** | | **Tu as déclaré son amour à Léa** | | **Tu as délégué ses tâches à Léa** | | **Tu as dispensé ses conseils à Léa** | | **Tu as donné sa chance à Léa** | | **Tu as écrit une lettre à Louis** | | **Tu as envoyé un baiser à Louis** | | **Tu as exposé ses raisons à Louis** | | **Tu as exprimé son amitié à Louis** | | **Tu as jeté un sort à Louis** | | **Tu as lancé une idée à Louis** | | **Tu as présenté sa démission à Louis** | | **Tu as raconté une histoire à Louis** | | **Tu as transféré la responsabilité à Louis** | | **Tu as transmis les consignes à Louis** | | **Louis t'a adressé un courrier** | | **Louis t'a cédé sa place** | | **Louis t'a chanté une chanson** | | **Louis t'a communiqué le message** | | **Louis t'a confié son secret** | | **Louis t'a consacré du temps** | | **Louis t'a déclaré son amour** | | **Louis t'a délégué ses tâches** | | **Louis t'a dispensé ses conseils** | | **Louis t'a donné une chance** | | **Léa t'a écrit une lettre** | | **Léa t'a envoyé un baiser** | | **Léa t'a exposé ses raisons** | | **Léa t'a exprimé son amitié** | | **Léa t'a jeté un sort** | | **Léa t'a lancé une idée** | | **Léa t'a présenté sa démission** | | **Léa t'a raconté une histoire** | | **Léa t'a transféré la responsabilité** | | **Léa t'a transmis les consignes** | | **Tu as adressé une vague à Léa** | | **Tu as cédé son nez à Léa** | | **Tu as chanté une cuisine à Léa** | | **Tu as communiqué la prison à Léa** | | **Tu as confié ses seuils à Léa** | | **Tu as consacré du tempête à Léa** | | **Tu as déclaré son mouvement à Léa** | | **Tu as délégué ses phrases à Léa** | | **Tu as dispensé ses villages à Léa** | | **Tu as donné une race à Léa** | | **Tu as écrit une viande à Louis** | | **Tu as envoyé un genou à Louis** | | **Tu as exposé ses maisons à Louis** | | **Tu as exprimé sa chemise à Louis** | | **Tu as jeté un cinéma à Louis** | | **Tu as lancé une église à Louis** | | **Tu as présenté sa cheminée à Louis** | | **Tu as raconté une fille à Louis** | | **Tu as transféré la boussole à Louis** | | **Tu as transmis les résidents à Louis** | | **Louis t'a adressé une vague** | | **Louis t'a cédé son nez** | | **Louis t'a chanté une cuisine** | | **Louis t'a communiqué la prison** | | **Louis t'a confié ses seuils** | | **Louis t'a consacré du tempête** | | **Louis t'a déclaré son mouvement** | | **Louis t'a délégué ses phrases** | | **Louis t'a dispensé ses villages** | | **Louis t'a donné une race** | | **Léa t'a écrit une viande** | | **Léa t'a envoyé un genou** | | **Léa t'a exposé ses maisons** | | **Léa t'a exprimé sa chemise** | | **Léa t'a jeté un cinéma** | | **Léa t'a lancé une église** | | **Léa t'a présenté sa cheminée** | | **Léa t'a raconté une fille** | | **Léa t'a transféré la boussole** | | **Léa t'a transmis les résidents** | | **Tu as apporté une bouteille à Louis** | | **Tu as assené un coup à Louis** | | **Tu as attribué une médaille à Louis** | | **Tu as confié la clé à Louis** | | **Tu as consigné un message à Louis** | | **Tu as donné un livre à Louis** | | **Tu as envoyé un paquet à Louis** | | **Tu as fourni les draps à Louis** | | **Tu as lancé la balle à Louis** | | **Tu as légué son appartement à Louis** | | **Tu as livré une pizza à Léa** | | **Tu as montré une photo à Léa** | | **Tu as offert un cadeau à Léa** | | **Tu as passé le plateau à Léa** | | **Tu as porté des fleurs à Léa** | | **Tu as prêté sa voiture à Léa** | | **Tu as rendu sa veste à Léa** | | **Tu as servi du thé à Léa** | | **Tu as vendu des cigarettes à Léa** | | **Tu as versé de l'eau à Léa** | | **Léa t'a apporté une bouteille** | | **Léa t'a assené un coup** | | **Léa t'a attribué une médaille** | | **Léa t'a confié la clé** | | **Léa t'a consigné un message** | | **Léa t'a donné un livre** | | **Léa t'a envoyé un paquet** | | **Léa t'a fourni les draps** | | **Léa t'a lancé la balle** | | **Léa t'a légué son appartement** | | **Louis t'a livré une pizza** | | **Louis t'a montré une photo** | | **Louis t'a offert un cadeau** | | **Louis t'a passé le plateau** | | **Louis t'a porté des fleurs** | | **Louis t'a prêté sa voiture** | | **Louis t'a rendu sa veste** | | **Louis t'a servi du thé** | | **Louis t'a vendu des cigarettes** | | **Louis t'a versé de l'eau** | | **Tu as apporté une nation à Léa** | | **Tu as assené un canard à Léa** | | **Tu as attribué une honte à Léa** | | **Tu as confié la cohérence à Léa** | | **Tu as consigné un retard à Léa** | | **Tu as donné un livre à Léa** | | **Tu as envoyé un appartement àLéa** | | **Tu as fourni la lune à Léa** | | **Tu as lancé le crocodile à Léa** | | **Tu as légué sa nature à Léa** | | **Tu as livré un ciel à Louis** | | **Tu as montré un rêve à Louis** | | **Tu as offert une conscience à Louis** | | **Tu as passé la bouche à Louis** | | **Tu as porté des déserts à Louis** | | **Tu as prêté sa naissance à Louis** | | **Tu as rendu son courage à Louis** | | **Tu as servi du remord à Louis** | | **Tu as vendu des doutes à Louis** | | **Tu as versé de l'obéissance à Louis** | | **Léa t'a apporté une nation** | | **Léa t'a assené un canard** | | **Léa t'a attribué une honte** | | **Léa t'a confié la cohérence** | | **Léa t'a consigné un retard** | | **Léa t'a donné une intelligence** | | **Léa t'a envoyé un appartement** | | **Léa t'a fourni la lune** | | **Léa t'a lancé le crocodile** | | **Léa t'a légué sa nature** | | **Louis t'a livré un ciel** | | **Louis t'a montré un rêve** | | **Louis t'a offert une conscience** | | **Louis t'a passé la bouche** | | **Louis t'a porté des déserts** | | **Louis t'a prêté sa naissance** | | **Louis t'a rendu son courage** | | **Louis t'a servi du remord** | | **Louis t'a vendu des doutes** | | **Louis t'a versé de l'obéissance** | |
| --- | --- | --- | --- | --- | --- | --- | --- | --- | --- | --- | --- | --- | --- | --- | --- | --- | --- | --- | --- | --- | --- | --- | --- | --- | --- | --- | --- | --- | --- | --- | --- | --- | --- | --- | --- | --- | --- | --- | --- | --- | --- | --- | --- | --- | --- | --- | --- | --- | --- | --- | --- | --- | --- | --- | --- | --- | --- | --- | --- | --- | --- | --- | --- | --- | --- | --- | --- | --- | --- | --- | --- | --- | --- | --- | --- | --- | --- | --- | --- | --- | --- | --- | --- | --- | --- | --- | --- | --- | --- | --- | --- | --- | --- | --- | --- | --- | --- | --- | --- | --- | --- | --- | --- | --- | --- | --- | --- | --- | --- | --- | --- | --- | --- | --- | --- | --- | --- | --- | --- | --- | --- | --- | --- | --- | --- | --- | --- | --- | --- | --- | --- | --- | --- | --- | --- | --- | --- | --- | --- | --- | --- | --- | --- | --- | --- | --- | --- | --- | --- | --- | --- | --- | --- | --- | --- | --- | --- | --- | --- | --- | --- | --- | --- | --- | --- | --- | --- | --- | --- | --- | --- | --- | --- | --- | --- | --- | --- | --- | --- | --- | --- | --- | --- | --- | --- | --- | --- | --- | --- | --- | --- | --- | --- | --- | --- | --- | --- | --- | --- | --- | --- | --- | --- | --- | --- | --- | --- | --- | --- | --- | --- | --- | --- | --- | --- | --- | --- | --- | --- | --- | --- | --- | --- | --- | --- | --- | --- | --- | --- | --- | --- | --- | --- | --- | --- | --- | --- | --- | --- | --- | --- | --- | --- | --- | --- | --- | --- | --- | --- | --- | --- | --- | --- | --- | --- | --- | --- | --- | --- | --- | --- | --- | --- | --- | --- | --- | --- | --- | --- | --- | --- | --- | --- | --- | --- | --- | --- | --- | --- | --- | --- | --- | --- | --- | --- | --- | --- | --- | --- | --- | --- | --- | --- | --- | --- | --- | --- | --- | --- | --- | --- | --- | --- | --- | --- | --- | --- | --- | --- | --- | --- | --- | --- | --- | --- | --- | --- | --- | --- | --- | --- |
