# Supplementary material for: The Agent is Right: When Motor Embodied Cognition is Space-Dependent
Source: PLoS One. 2011 Sep 23;6(9):e25036. doi: 10.1371/journal.pone.0025036 (PMC3179480; doi:10.1371/journal.pone.0025036)
Supplement: Appendix S2 — Set of stimuli used in experiment 2 , 3 and 4 . (DOC) [file pone.0025036.s002.doc]

| | **1** | | --- | | **2** | | **3** | | **4** | | **5** | | **6** | | **7** | | **8** | | **9** | | **10** | | **11** | | **12** | | **13** | | **14** | | **15** | | **16** | | **17** | | **18** | | **19** | | **20** | | **21** | | **22** | | **23** | | **24** | | **25** | | **26** | | **27** | | **28** | | **29** | | **30** | | **31** | | **32** | | **33** | | **34** | | **35** | | **36** | | **37** | | **38** | | **39** | | **40** | | **41** | | **42** | | **43** | | **44** | | **45** | | **46** | | **47** | | **48** | | **49** | | **50** | | **51** | | **52** | | **53** | | **54** | | **55** | | **56** | | **57** | | **58** | | **59** | | **60** | | **61** | | **62** | | **63** | | **64** | | **65** | | **66** | | **67** | | **68** | | **69** | | **70** | | **71** | | **72** | | **73** | | **74** | | **75** | | **76** | | **77** | | **78** | | **79** | | **80** | | **81** | | **82** | | **83** | | **84** | | **85** | | **86** | | **87** | | **88** | | **89** | | **90** | | **91** | | **92** | | **93** | | **94** | | **95** | | **96** | | **97** | | **98** | | **99** | | **100** | | **101** | | **102** | | **103** | | **104** | | **105** | | **106** | | **107** | | **108** | | **109** | | **110** | | **111** | | **112** | | **113** | | **114** | | **115** | | **116** | | **117** | | **118** | | **119** | | **120** | | **121** | | **122** | | **123** | | **124** | | **125** | | **126** | | **127** | | **128** | | **129** | | **130** | | **131** | | **132** | | **133** | | **134** | | **135** | | **136** | | **137** | | **138** | | **139** | | **140** | | **141** | | **142** | | **143** | | **144** | | **145** | | **146** | | **147** | | **148** | | **149** | | **150** | | **151** | | **152** | | **153** | | **154** | | **155** | | **156** | | **157** | | **158** | | **159** | | **160** | | | **Louis a adressé un courrier à Léa** |  | | --- | --- | | **Louis a cédé sa place à Léa** |  | | **Louis a chanté une chanson à Léa** |  | | **Louis a communiqué le message à Léa** |  | | **Louis a confié son secret à Léa** |  | | **Louis a consacré du temps à Léa** |  | | **Louis a déclaré son amour à Léa** |  | | **Louis a délégué ses tâches à Léa** |  | | **Louis a dispensé ses conseils à Léa** |  | | **Louis a donné une chance à Léa** |  | | **Louis a écrit une lettre à Léa** |  | | **Louis a envoyé un baiser à Léa** |  | | **Louis a exposé ses raisons à Léa** |  | | **Louis a exprimé son amitié à Léa** |  | | **Louis a jeté un sort à Léa** |  | | **Louis a lancé une idée à Léa** |  | | **Louis a présenté sa démission à Léa** |  | | **Louis a raconté une histoire à Léa** |  | | **Louis a transféré la responsabilité à Léa** |  | | **Louis a transmis les consignes à Léa** |  | | **Léa a adressé un courrier à Louis** |  | | **Léa a cédé sa place à Louis** |  | | **Léa a chanté une chanson à Louis** |  | | **Léa a communiqué le message à Louis** |  | | **Léa a confié son secret à Louis** |  | | **Léa a consacré du temps à Louis** |  | | **Léa a déclaré son amour à Louis** |  | | **Léa a délégué ses tâches à Louis** |  | | **Léa a dispensé ses conseils à Louis** |  | | **Léa a donné une chance à Louis** |  | | **Léa a écrit une lettre à Louis** |  | | **Léa a envoyé un baiser à Louis** |  | | **Léa a exposé ses raisons à Louis** |  | | **Léa a exprimé son amitié à Louis** |  | | **Léa a jeté un sort à Louis** |  | | **Léa a lancé une idée à Louis** |  | | **Léa a présenté sa démission à Louis** |  | | **Léa a raconté une histoire à Louis** |  | | **Léa a transféré la responsabilité à Louis** |  | | **Léa a transmis les consignes à Louis** |  | | **Louis a adressé une vague à Léa** |  | | **Louis a cédé son nez à Léa** |  | | **Louis a chanté une cuisine à Léa** |  | | **Louis a communiqué la prison à Léa** |  | | **Louis a confié ses seuils à Léa** |  | | **Louis a consacré du tempête à Léa** |  | | **Louis a déclaré son mouvement à Léa** |  | | **Louis a délégué ses phrases à Léa** |  | | **Louis a dispensé ses villages à Léa** |  | | **Louis a donné une race à Léa** |  | | **Louis a écrit une viande à Léa** |  | | **Louis a envoyé un genou à Léa** |  | | **Louis a exposé ses maisons à Léa** |  | | **Louis a exprimé sa chemise à Léa** |  | | **Louis a jeté un cinéma à Léa** |  | | **Louis a lancé une église à Léa** |  | | **Louis a présenté sa cheminée à Léa** |  | | **Louis a raconté une fille à Léa** |  | | **Louis a transféré la boussole à Léa** |  | | **Louis a transmis les résidents à Léa** |  | | **Léa a adressé une vague à Louis** |  | | **Léa a cédé son nez à Louis** |  | | **Léa a chanté une cuisine à Louis** |  | | **Léa a communiqué la prison à Louis** |  | | **Léa a confié ses seuils à Louis** |  | | **Léa a consacré du tempête à Louis** |  | | **Léa a déclaré son mouvement à Louis** |  | | **Léa a délégué ses phrases à Louis** |  | | **Léa a dispensé ses villages à Louis** |  | | **Léa a donné une race à Louis** |  | | **Léa a écrit une viande à Louis** |  | | **Léa a envoyé un genou à Louis** |  | | **Léa a exposé ses maisons à Louis** |  | | **Léa a exprimé sa chemise à Louis** |  | | **Léa a jeté un cinéma à Louis** |  | | **Léa a lancé une église à Louis** |  | | **Léa a présenté sa cheminée à Louis** |  | | **Léa a raconté une fille à Louis** |  | | **Léa a transféré la boussole à Louis** |  | | **Léa a transmis les résidents à Louis** |  | | **Louis a apporté une bouteille à Léa** |  | | **Louis a assené un coup à Léa** |  | | **Louis a attribué une médaille à Léa** |  | | **Louis a confié la clé à Léa** |  | | **Louis a consigné un message à Léa** |  | | **Louis a donné un livre à Léa** |  | | **Louis a envoyé un paquet à Léa** |  | | **Louis a fourni les draps à Léa** |  | | **Louis a lancé la balle à Léa** |  | | **Louis a légué son appartement à Léa** |  | | **Louis a livré une pizza à Léa** |  | | **Louis a montré une photo à Léa** |  | | **Louis a offert un cadeau à Léa** |  | | **Louis a passé le plateau à Léa** |  | | **Louis a porté des fleurs à Léa** |  | | **Louis a prêté sa voiture à Léa** |  | | **Louis a rendu sa veste à Léa** |  | | **Louis a servi du thé à Léa** |  | | **Louis a vendu des cigarettes à Léa** |  | | **Louis a versé de l'eau à Léa** |  | | **Léa a apporté une bouteille à Louis** |  | | **Léa a assené un coup à Louis** |  | | **Léa a attribué une médaille à Louis** |  | | **Léa a confié la clé à Louis** |  | | **Léa a consigné un message à Louis** |  | | **Léa a donné un livre à Louis** |  | | **Léa a envoyé un paquet à Louis** |  | | **Léa a fourni les draps à Louis** |  | | **Léa a lancé la balle à Louis** |  | | **Léa a légué son appartement à Louis** |  | | **Léa a livré une pizza à Louis** |  | | **Léa a montré une photo à Louis** |  | | **Léa a offert un cadeau à Louis** |  | | **Léa a passé le plateau à Louis** |  | | **Léa a porté des fleurs à Louis** |  | | **Léa a prêté sa voiture à Louis** |  | | **Léa a rendu sa veste à Louis** |  | | **Léa a servi du thé à Louis** |  | | **Léa a vendu des cigarettes à Louis** |  | | **Léa a versé de l'eau à Louis** |  | | **Louis a apporté une nation à Léa** |  | | **Louis a assené un canard à Léa** |  | | **Louis a attribué une honte à Léa** |  | | **Louis a confié la cohérence à Léa** |  | | **Louis a consigné un retard à Léa** |  | | **Louis a donné un livre à Léa** |  | | **Louis a envoyé un appartement à Léa** |  | | **Louis a fourni la lune à Léa** |  | | **Louis a lancé le crocodile à Léa** |  | | **Louis a légué sa nature à Léa** |  | | **Louis a livré un ciel à Léa** |  | | **Louis a montré un rêve à Léa** |  | | **Louis a offert une conscience à Léa** |  | | **Louis a passé la bouche à Léa** |  | | **Louis a porté des déserts à Léa** |  | | **Louis a prêté sa naissance à Léa** |  | | **Louis a rendu son courage à Léa** |  | | **Louis a servi du remord à Léa** |  | | **Louis a vendu des doutes à Léa** |  | | **Louis a versé de l'obéissance à Léa** |  | | **Léa a apporté une nation à Louis** |  | | **Léa a assené un canard à Louis** |  | | **Léa a attribué une honte à Louis** |  | | **Léa a confié la cohérence à Louis** |  | | **Léa a consigné un retard à Louis** |  | | **Léa a donné une intelligence à Louis** |  | | **Léa a envoyé un appartement à Louis** |  | | **Léa a fourni la lune à Louis** |  | | **Léa a lancé le crocodile à Louis** |  | | **Léa a légué sa nature à Louis** |  | | **Léa a livré un ciel à Louis** |  | | **Léa a montré un rêve à Louis** |  | | **Léa a offert une conscience à Louis** |  | | **Léa a passé la bouche à Louis** |  | | **Léa a porté des déserts à Louis** |  | | **Léa a prêté sa naissance à Louis** |  | | **Léa a rendu son courage à Louis** |  | | **Léa a servi du remord à Louis** |  | | **Léa a vendu des doutes à Louis** |  | | **Léa a versé de l'obéissance à Louis** |  | |
| --- | --- | --- | --- | --- | --- | --- | --- | --- | --- | --- | --- | --- | --- | --- | --- | --- | --- | --- | --- | --- | --- | --- | --- | --- | --- | --- | --- | --- | --- | --- | --- | --- | --- | --- | --- | --- | --- | --- | --- | --- | --- | --- | --- | --- | --- | --- | --- | --- | --- | --- | --- | --- | --- | --- | --- | --- | --- | --- | --- | --- | --- | --- | --- | --- | --- | --- | --- | --- | --- | --- | --- | --- | --- | --- | --- | --- | --- | --- | --- | --- | --- | --- | --- | --- | --- | --- | --- | --- | --- | --- | --- | --- | --- | --- | --- | --- | --- | --- | --- | --- | --- | --- | --- | --- | --- | --- | --- | --- | --- | --- | --- | --- | --- | --- | --- | --- | --- | --- | --- | --- | --- | --- | --- | --- | --- | --- | --- | --- | --- | --- | --- | --- | --- | --- | --- | --- | --- | --- | --- | --- | --- | --- | --- | --- | --- | --- | --- | --- | --- | --- | --- | --- | --- | --- | --- | --- | --- | --- | --- | --- | --- | --- | --- | --- | --- | --- | --- | --- | --- | --- | --- | --- | --- | --- | --- | --- | --- | --- | --- | --- | --- | --- | --- | --- | --- | --- | --- | --- | --- | --- | --- | --- | --- | --- | --- | --- | --- | --- | --- | --- | --- | --- | --- | --- | --- | --- | --- | --- | --- | --- | --- | --- | --- | --- | --- | --- | --- | --- | --- | --- | --- | --- | --- | --- | --- | --- | --- | --- | --- | --- | --- | --- | --- | --- | --- | --- | --- | --- | --- | --- | --- | --- | --- | --- | --- | --- | --- | --- | --- | --- | --- | --- | --- | --- | --- | --- | --- | --- | --- | --- | --- | --- | --- | --- | --- | --- | --- | --- | --- | --- | --- | --- | --- | --- | --- | --- | --- | --- | --- | --- | --- | --- | --- | --- | --- | --- | --- | --- | --- | --- | --- | --- | --- | --- | --- | --- | --- | --- | --- | --- | --- | --- | --- | --- | --- | --- | --- | --- | --- | --- | --- | --- | --- | --- | --- | --- | --- | --- | --- | --- | --- | --- | --- | --- | --- | --- | --- | --- | --- | --- | --- | --- | --- | --- | --- | --- | --- | --- | --- | --- | --- | --- | --- | --- | --- | --- | --- | --- | --- | --- | --- | --- | --- | --- | --- | --- | --- | --- | --- | --- | --- | --- | --- | --- | --- | --- | --- | --- | --- | --- | --- | --- | --- | --- | --- | --- | --- | --- | --- | --- | --- | --- | --- | --- | --- | --- | --- | --- | --- | --- | --- | --- | --- | --- | --- | --- | --- | --- | --- | --- | --- | --- | --- | --- | --- | --- | --- | --- | --- | --- | --- | --- | --- | --- | --- | --- | --- | --- | --- | --- | --- | --- | --- | --- | --- | --- | --- | --- | --- | --- | --- | --- | --- | --- | --- | --- | --- | --- | --- | --- | --- | --- | --- | --- | --- | --- | --- | --- | --- | --- | --- | --- | --- | --- | --- | --- | --- | --- | --- | --- | --- | --- | --- | --- | --- | --- | --- | --- | --- | --- | --- | --- | --- | --- | --- | --- | --- | --- | --- | --- | --- |
