# Supplementary material for: The Agent is Right: When Motor Embodied Cognition is Space-Dependent
Source: PLoS One. 2011 Sep 23;6(9):e25036. doi: 10.1371/journal.pone.0025036 (PMC3179480; doi:10.1371/journal.pone.0025036)
Supplement: Appendix S3 — Example of questionnaire used for debriefing after experiments 2 , 3 and 4 . (DOC) [file pone.0025036.s003.doc]

Questionnaire

Vous devrez indiquer sur la ligne horizontale votre degré d’accord avec chaque affirmation, sachant que l’extrême gauche correspond à «Pas du tout d’accord», l’extrême droite correspond à «Tout à fait d’accord», un trait au milieu de la ligne correspond à « Ni d’accord ni pas d’accord ».

*Please indicate on the horizontal line your level of agreement with each statement, knowing that the extreme left is “I do not agree at all”, the extreme right corresponds to "I totally agree", a trait in the middle of the line corresponds to "neither agree nor disagree."*

1. La tâche était facile à comprendre

*The task was easy to understand*

1. J’ai bien compris qu’elle était ma perspective

*I fully understood what my perspective was*

1. Il me semblait difficile de pouvoir faire des mouvements comme si j’étais Léa

*It seemed difficult to me to make movements as if I was Léa/Louis*

1. Il a été très facile de faire des mouvements comme si j’étais Léa

*It was really easy for me to do movements as if I was Léa/Louis*

1. J’étais plus attentif-ive au sens des phrases qu’à l’interaction qu’elles décrivent

*I paid more attention to the meaning of sentences than to the interactions they described*

1. Il a été plus facile de prendre la perspective de Léa quand elle était à gauche

*It was easier to take Léa/Louis’s perspective when he/she was on the left*

1. Je me suis immédiatement sentie à la place de Léa

*I immediately felt like being Léa/Louis*

1. L’expérience s’est bien passée

*The experiment went well*

1. J’avais des difficultés à prendre le point de vue de Léa

*I had difficulties in taking the perspective of Léa/Louis*

1. J’étais attentif-ive à la relation entre les deux personnages

*I paid a lot of attention to the relationship between the two character*

1. Le point de vue de Léa est devenu très vite le mien

*The point of view of Léa/Louis quickly became mine*

1. J’ai trouvé que mon mouvement était influencé par le mouvement décrit dans les phrases

*I found that my movement was influenced by the movement described in the sentences*

1. Je ne me sentais jamais vraiment à la place de Léa

*I never felt to be truly in Léa/Louis’ shoes*

1. J’étais concentré sur l’évaluation des phrases

*I focused on sentence evaluation*

1. Je me sentais sans émotion par rapport à la situation de Léa

*I had no emotions with respect to Léa/Louis situation*

1. J’avais des difficultés à comprendre si la phrase était plausible ou pas

*I had difficulties to understand whether the sentences made sense or not*

1. Quand Léa était à droite, il a été plus facile de prendre sa perspective

*When Léa/Louis was right, it was easier to take her/his perspective*

1. J’avais l’impression d’avoir des émotions comme si j’étais Léa

*I felt like I had emotions like Léa/Louis*
