# Supplementary material for: The Agent is Right: When Motor Embodied Cognition is Space-Dependent
Source: PLoS One. 2011 Sep 23;6(9):e25036. doi: 10.1371/journal.pone.0025036 (PMC3179480; doi:10.1371/journal.pone.0025036)
Supplement: Appendix S4 — Separate performances for abstract and concrete sentences in all experiments. (DOC) [file pone.0025036.s004.doc]

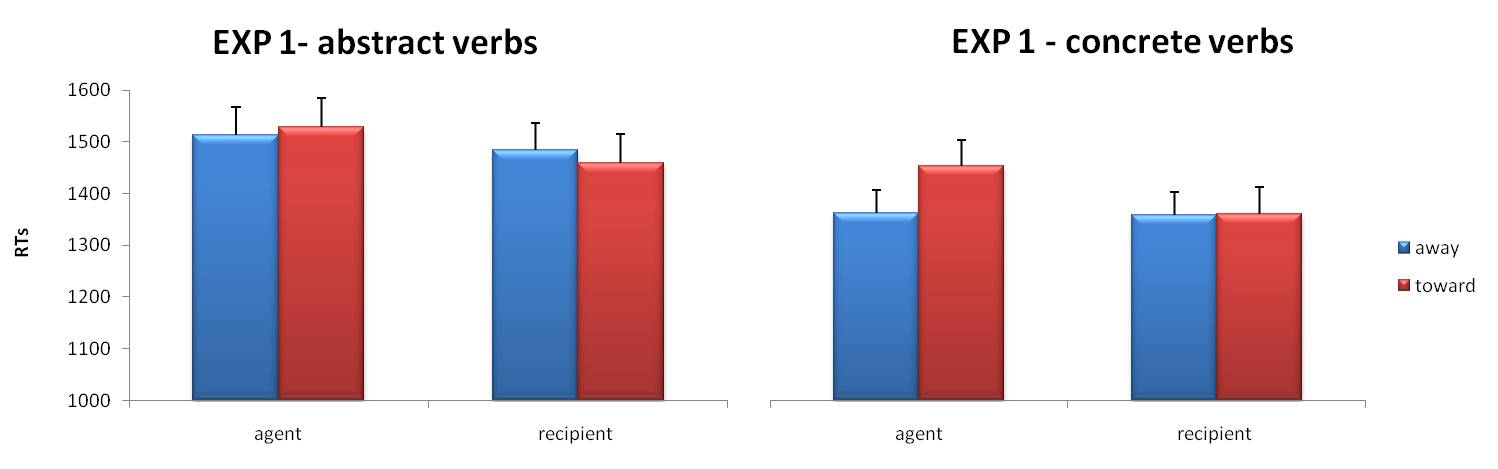
fig. a – Mean RTs as a function of the variable Role and Movement in experiment 1 for abstract and concrete verbs
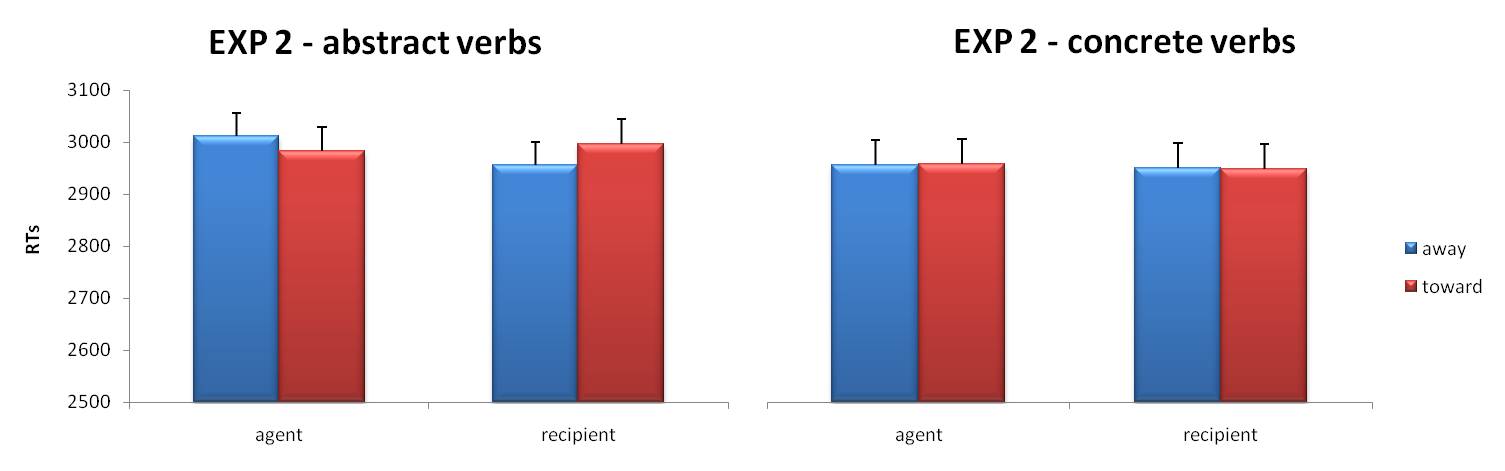


fig. b – Mean RTs as a function of the variable Role and Movement in experiment 2 for abstract and concrete verbs
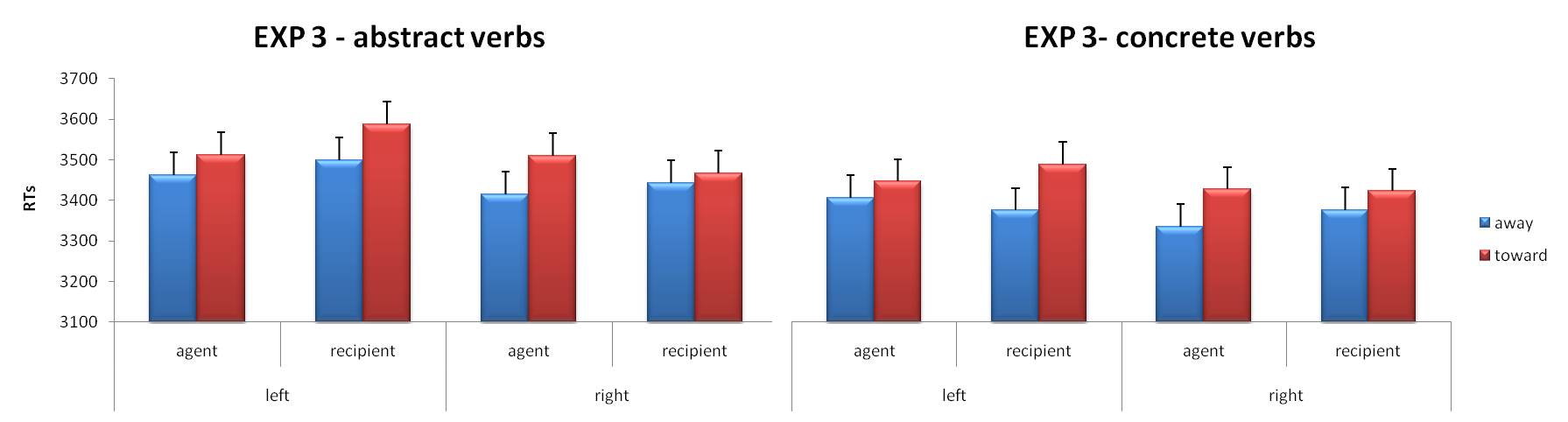
fig. c – Mean RTs as a function of the variable Role and Movement in experiment 3 for abstract and concrete verbs


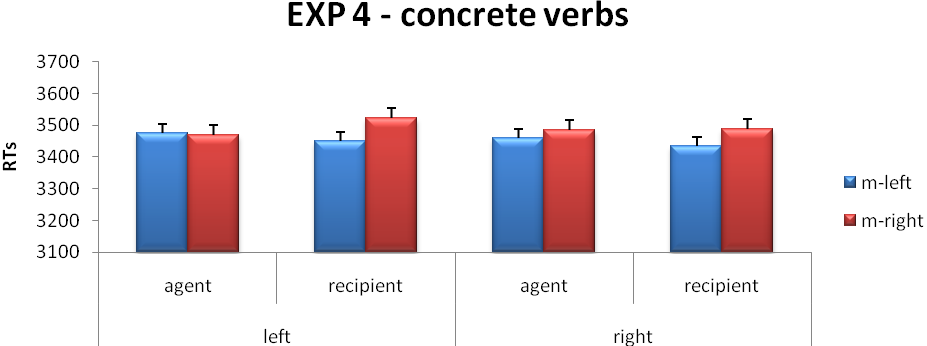


fig. d – Mean RTs as a function of the variable Role and Movement in experiment 4 for concrete verbs only (N = 42)


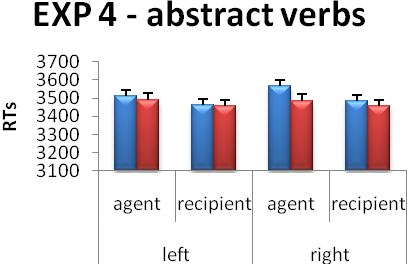

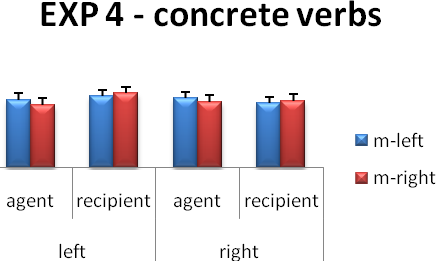


fig. e – Mean RTs as a function of the variable Role and Movement in experiment 4 for abstract and concrete verbs (N=18)
