# Supplementary material for: The Agent is Right: When Motor Embodied Cognition is Space-Dependent
Source: PLoS One. 2011 Sep 23;6(9):e25036. doi: 10.1371/journal.pone.0025036 (PMC3179480; doi:10.1371/journal.pone.0025036)
Supplement: Appendix S5 — Translation of complete instructions for experiment 2 . (DOC) [file pone.0025036.s005.doc]

Instructions for the half of the experiment in which the sensibility was coupled with backward movement direction:

*“During this experiment, phrases describing an interaction between two people, Lea and Louis, will be presented to you. These sentences may make sense or not.*

*You are asked to carefully read these sentences and to respond with a joystick movement whether they make sense or not. If a sentence is plausible, make a backward movement. If not, make a forward movement.*

*During this experiment we ask you to put into Léa’s shoes while reading these sentences and so to take her point of view.*

*Try to be as fast and as precise as you can. A training phase will be followed by 4 blocks with a pause before each block. “*

The instructions for the half of the experiment in which the sensibility was coupled with forward movement direction:

*“During this experiment, phrases describing an interaction between two people, Lea and Louis, will be presented to you. These sentences may make sense or not.*

*You are asked to carefully read these sentences and to respond with a joystick movement whether they make sense or not. If a sentence is plausible, make a forward movement. If not, make a backward movement.*

*During this experiment we ask you to put into Léa’s shoes while reading these sentences and so to take her point of view.*

*Try to be as fast and as precise as you can. A training phase will be followed by 4 blocks with a pause before each block. “*
